# An Efficient Procedure Based on a MW-Assisted Horner–Wadsworth-Emmons Reaction for the Synthesis of (*Z*)-3,3-Trisubstituted-α,β-unsaturated Esters 

**DOI:** 10.3390/molecules15095928

**Published:** 2010-08-27

**Authors:** Daniela Rossi, Anna Carnevale Baraglia, Massimo Serra, Ornella Azzolina, Simona Collina

**Affiliations:** Dipartimento di Chimica Farmaceutica, viale Taramelli 12, Pavia 27100, Italy

**Keywords:** (*Z*)-3,3-trisubstituted α,β-unsaturated methyl esters, aryl-alkyl ketones, Horner–Wadsworth-Emmons reaction, microwave-assisted reactions

## Abstract

A microwave-assisted HWE olefination process of readily accessible aryl-alkyl ketones has been developed to provide a rapid access to (*Z*)-3,3-trisubstituted-α,β-unsaturated methyl esters, key building blocks for the synthesis of biologically active compounds.

## 1. Introduction

Recently, as part of our research program on new classes of compounds potentially active in the treatment of neurodegenerative diseases, we focused on the design and synthesis of novel PKC ligands. To achieve this goal and to investigate the molecular features involved in PKC binding, a drug discovery library based on a 3-aryl-4-hydroxybutyrate scaffold (Figure 1) was designed [1].

**Figure 1:**
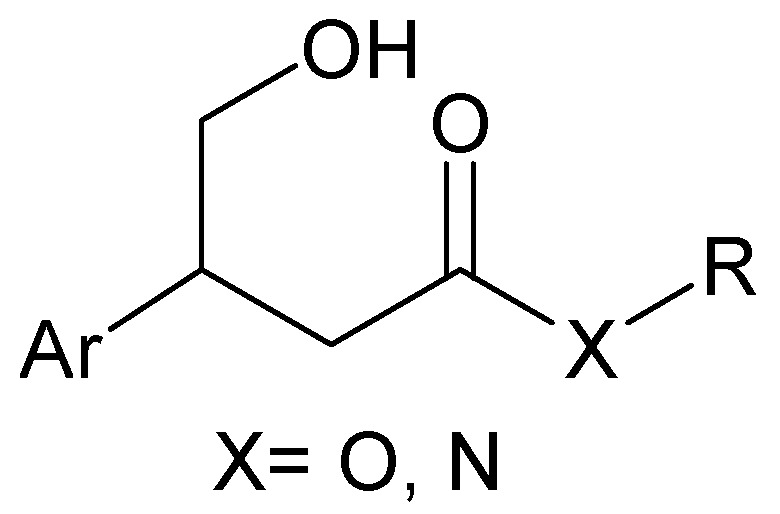
3-Aryl-4-hydroxybutyrate scaffold.

**Scheme 1:**
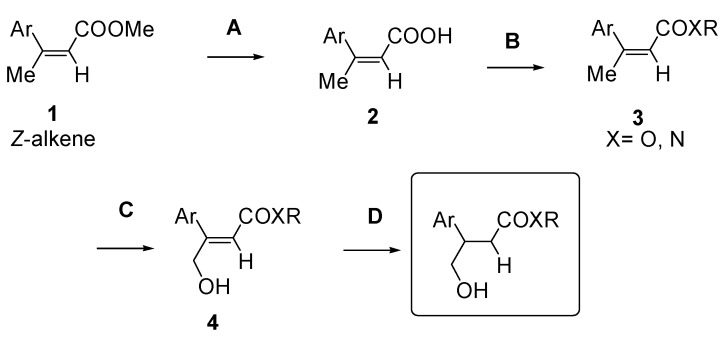
Proposed synthetic pathway for the preparation of the 3-aryl-4-hydroxybutyrate scaffold.

3,3-Trisubstituted α,β-unsaturated methyl esters **1** were identified as valuable synthetic intermediates to achieve, after simple synthetic elaborations, the desired chemical diversity of the library (Scheme 1). It has to be outlined that the allylic oxidation step of the proposed synthetic pathway (Scheme 1, step C) is selective toward α,β-unsaturated esters with (*Z*)-configuration [2]. Accordingly, our principal aim was to come up with a rapid and economic synthetic plan for the preparation of (*Z*)-3,3-trisubstituted-α,β-unsaturated methyl esters in amounts suitable for their subsequent derivatization and for preparing the whole compound library.

A well known method for the synthesis of disubstituted olefins is the Horner–Wadsworth-Emmons (HWE) reaction. The (*E*)- or (*Z*)- geometrical selectivity of HWE olefination of aldehydes has been extensively studied under conventional conditions [3] and only recently under microwave irradiation [4]. Only a few papers on HWE olefination of ketones have been described under conventional conditions [5] and, to the best of our knowledge, the effect of microwave heating has never been studied.

Techniques used for the preparation of compounds collections have evolved very rapidly over the past two decades. Emphasis was initially placed on the development of solid-phase and solution-phase synthetic methods [6,7]. In recent years, the development of combinatorial synthetic techniques using microwave irradiation was also emphasized [8]. The use of microwave heating has indeed greatly affected many aspects of chemical synthesis since it can dramatically reduce the reaction time, increase the product purity and yield and allow a strict control of the reaction parameters [9]. In this Communication we report a microwave-assisted HWE olefination process of readily accessible aryl-alkyl ketones, which quickly provides a small collection of (*Z*)-3,3-trisubstituted-α,β-unsaturated methyl esters in high efficiency. 

## 2. Results and Discussion

The first step of our work consisted in comparing conventional and microwave assisted synthesis of 3,3-trisubstituted-α,β-unsaturated methyl esters **1**. The procedure of Sano and co-workers was initially adopted employing bis(2,2,2-trifluoroethyl)(methoxycarbonylmethyl)-phosphonate (**5**) combined with stannous trifluoromethanesulfonate [(CF_3_SO_3_)_2_Sn] and *N*-ethylpiperidine in anhydrous dichloro-methane (DCM) [5]. The experimental conditions required two reaction steps, low temperatures and long reaction times. Generally, low yields and especially unsatisfactory reproducibility were evidenced. Consequently, microwave-assisted synthesis was tested on 50 mg of **6a** (1 equiv), using **5** (1.5 equiv), (CF_3_SO_3_)_2_Sn (1.7 equiv) and *N*-ethylpiperidine (1.5 equiv), in 3 mL of anhydrous DCM under microwave-induced heating at 70 °C for 30 min (Table 1, entry 1).

**Table 1:** Set up of the experimental conditions for microwave-promoted HWE olefination of **6a**.

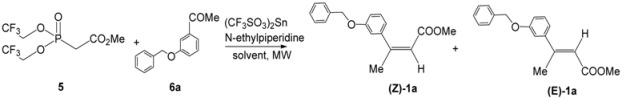						
Entry	Solvent	5 equiv	T (°C)	Time (min)	Conversion ^a ^(%)	(*Z*)/(*E*) ratio^a^
**1**	DCM	1.5	70	30	58	71:29
**2**	DCM	3	70	30	58	69:31
**3**	DCM	4.2	70	30	63	70:30
**4**	DMF	4.2	70	30	-	-
**5**	DMF	4.2	150	30	-	-
**6**	THF	4.2	70	30	-	-
**7**	THF	4.2	150	30	-	-
**8**	DCE	4.2	70	30	59	67:33
**9**	DCE	4.2	120	30	69	64:36
**10**	DCE	4.2	120	20	72	65:35
**11**	DCE	4.2	150	30	85	69:31
**12**	DCE	4.2	150	20	98	81:19
**13**	DCE	4.2	150	15	84	75:25
**14**	DCE	4.2	150	10	47	61:39
**15**	DCE	3	150	30	77	61:39
**16**	DCE	3	150	20	82	65:35
**17**	DCE	3	150	15	76	65:35
**18**	DCE	3	150	10	61	50:50

^a ^Determined on crude products by HPLC-UV/PAD analysis registered at 225 nm.

^1^H-NMR analysis of the crude **1a** evidenced the presence of both the (*Z*)- and (*E*)- isomers: two different signals corresponding to the olefinic hydrogen (5.95 ppm and 6.16 ppm), and two signals related to the methyl group on the double bond (2.20 ppm and 2.59 ppm) were present; the integration of the above-mentioned signals allowed us to determine that the ratio between these two species was about 70/30. The reaction, repeated three times, showed high reproducibility.

Both isomers were isolated from the reaction mixture via flash chromatography and characterized by ^1^H-NMR, ^13^C-NMR, NOESY, IR, and high performance liquid chromatography-ultraviolet photodiode array (HPLC-UV/PAD) analysis. Concerning NOESY experiments, a significant NOE effect corresponding to the H-H interaction between the methyl group on the double bond and the olefinic hydrogen was evidenced for both the isomers; nevertheless these effects resulted of different intensity, decreasing from the major isomer to the minor one. Accordingly, the (*Z*)-configuration was assigned to the major reaction product.

With the efficiency of the MW-assisted procedure proven, the second part of our work consisted in optimizing the reaction conditions in order to maximize both reaction geometrical selectivity and conversion of **6a** into the corresponding 3,3-trisubstituted-α,β-unsaturated methyl ester (Table 1). To easily determine the conversion percentage and the geometrical selectivity of the reaction during the set up of the experimental conditions, an opportune HPLC-UV/PAD method was developed. Pure (*Z*)-**1a** and (*E*)-**1a** were successfully analyzed employing a Intersil ACE-3 C_8_ column and water and acetonitrile as mobile phase. The retention times of (*Z*)-**1a** and (*E*)-**1a** were 5.5 min and 7.1 min (Rs = 3.02), respectively. It has to be noted that like other olefinic compounds [10,11], the (*Z)*- and (*E)*-isomers of **1a** showed different UV profiles (Figure 1). Therefore, various attempts were performed in order to identify the right wavelength for their quantification. The HPLC-UV/PAD analysis of crude **1a**, recorded at 225 nm, evidenced a reaction geometrical selectivity closed to 70% (Table 1, entry 1), according to the ^1^H-NMR analysis (Figure 2).

**Figure 1:**
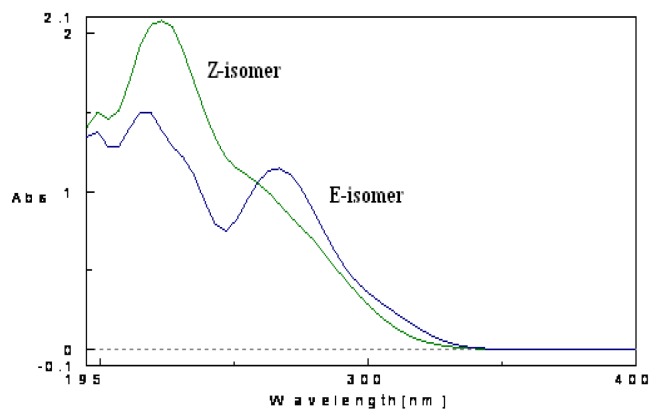
UV absorption spectra of compounds (*Z*)-**1a** and (*E*)-**1a**.

**Figure 2:**
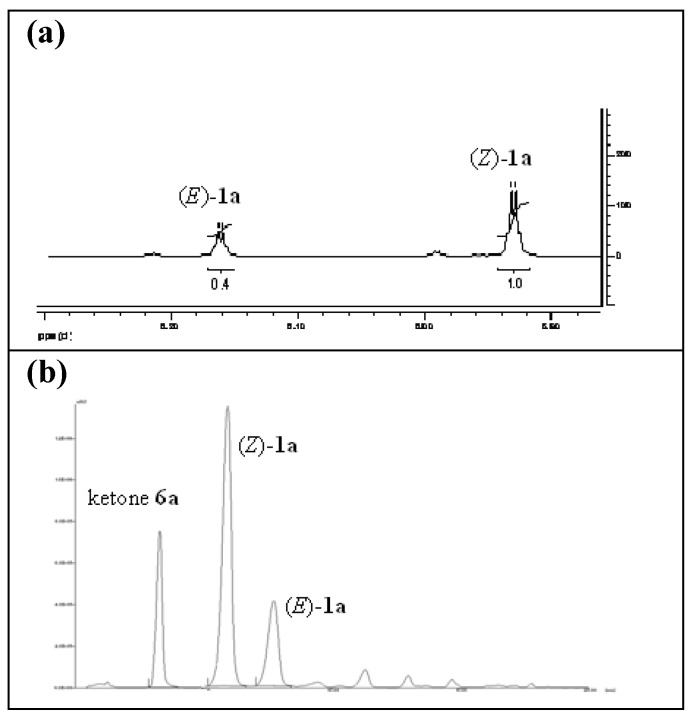
**(a)** NMR spectrum of crude **1a** ranging from δ = 5.80 ppm to δ = 6.25 ppm; **(b)** Chromatographic profile of crude **1a**.

Concerning the set up of the reaction conditions, the effect of reagent amounts was firstly investigated, keeping constant the reaction temperature at 70 °C and the reaction time at 30 min (Table 1, entries 1-3). The best results were achieved employing 4.2 equiv of **5** combined with 1.7 equiv of (CF_3_SO_3_)_2_Sn and 1.5 equiv of *N*-ethylpiperidine. Indeed, (*Z*)-**1a** was synthesized with conversion higher than 60% and selectivity closed to 70% (Table 1, entry 3). The HWE olefination of **6a** was also experimented in DCM at room temperature as well as under conventional heating (reflux). Compound **1a **was detected only in traces and the formation of several by-products was evidenced (TLC analysis). 

Encouraged by results obtained for the MW assisted HWE olefination of **6a**, further experiments were conducted using anhydrous dimethylformamide (DMF), dichloroethane (DCE) and tetrahydro-furan (THF), solvents commonly used in the HWE reaction, keeping constant the number of equivalents of **5** at 4.2 and varying both reaction time and temperature (Table 1, entries 4-14). Concerning MW absorber properties, THF is a low absorber (tan δ < 0.1) like DCM, while DMF and DCE are considered medium absorbers (tan δ between 0.1 and 0.5). Notably, employing DCE at 150 °C the reaction was driven to completion in 20 min (98% conversion) with a (*Z*)/(*E*) selectivity higher than 80% (Table 1, entry 12), whereas in THF and in DMF **1a** was detected only in traces. Therefore, chlorinated solvents seem to be necessary for the MW assisted HWE reaction.

Concerning the reaction time, the extension from 20 to 30 min significantly decreased both conversion and selectivity; similarly, reducing the run time from 20 to 15 and to 10 min a progressive decrease in conversion and reaction selectivity was evidenced. An analogous trend was observed when 3 equivalents of **5** were used (Table 1, entries 15-18), confirming that the optimal reaction time is 20 min. As regards to reaction temperature, a progressive decrease in the conversion was noticed when the temperature was decreased from 150 to 120 and to 70 °C. In summary, the optimized protocol involves microwave-induced heating of **6a** in anhydrous DCE for 20 min at 150 °C in the presence of **5** (4.2 equiv) combined with (CF_3_SO_3_)_2_Sn (1.7 equiv) and *N*-ethylpiperidine (1.5 equiv). Olefin **1a** was obtained with a conversion of 98% as a mixture of isomers (*Z*) / (*E*) 81:19 (Table 1, entry 12).

Finally, the optimized experimental conditions were extended to all substrates to prepare compounds **1b-h** (Table 2). Crude **1b-h** were analyzed using the above-described HPLC-UV/PAD method. A good resolution between the (*Z*)- and (*E*)-isomers was obtained for all compounds. Nevertheless, (Z)/(E) ratio could not be determined by HPLC analysis, due to the remarkable differences in the UV spectra of (Z) and (E) isomers (Appendix I). Briefly, various attempts were performed in order to identify the wavelength suitable to furnish a (Z)/(E) ratio in accordance with ^1^H-NMR analysis, but unsatisfactory results were obtained. Therefore the conversion percentage and the isomeric ratio were determined by^ 1^H-NMR analysis of the crude products.

As stated in Table 2, all compounds were obtained with a conversion higher than 55%. As regards to the geometrical selectivity of the reaction, a satisfactory (*Z*)- selectivity (closed to 70%) was evidenced for all prepared compounds, with the only exception of compound **1g** (Table 2, entry 6), suggesting the good effect of microwave heating on the stereoselective coupling of various aryl-methyl ketones with bis(2,2,2-trifluoroethyl)-phosphonoester via HWE reaction.

**Table 2:** HWE olefination of aryl methyl ketones **6b-h**.

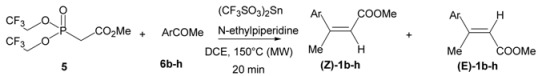				
Entry	Ar	Olefin	Conversion (%)^a^	(*Z*)/(*E*)ratio^a^
**1**	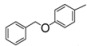	**1b**	55	66:34
**2**	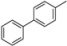	**1c**	99	74:26
**3**	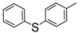	**1d**	86	76:24
**4**	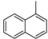	**1e**	58	79:21
**5**	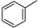	**1f**	92	66:34
**6**	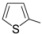	**1g**	56	61:39
**7**	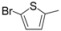	**1h**	60	74:26

^a.^Determined on crude products by 400-MHz ^1^H-NMR.

## 3. Experimental

### 3.1. General

Unless otherwise specified, commercially available reagents were used as received from the supplier. Solvents were purified according to the guidelines in Purification of Laboratory Chemicals [12]. All MW reactions were performed in a CEM Corporation Discover^®^ LabMate, equipped with an IR probe. Melting points were measured on SMP3 Stuart Scientific apparatus and are uncorrected. Analytical TLC were carried out on silica gel precoated glass-backed plates (Fluka Kieselgel 60 F254, Merck) and visualized by ultra-violet radiation, acidic ammonium molybdate (IV) or potassium permanganate. Flash SiO_2_ column chromatographies were performed with a Biotage High-Performance Flash Chromatography system with 12-mm or 25-mm flash cartridges. ^1^H-NMR spectra were measured with a Bruker AVANCE 400 spectrometer (Germany) at room temperature. Chemical shifts are given in ppm (δ), coupling constants (*J*) are in Hertz (Hz) and signals are designated as follows: (s) singlet, (d) doublet, (t) triplet, (q) quartet, (br s) broad singlet, and (m) multiplet. TMS was used as internal standard. Elemental analyses (C, H, N) were performed on a Carlo Erba 1106 analyzer and the analysis results were found to be in good agreement ( ± 0.2%) with the theoretical values. HPLC-UV/PAD analyses were performed on a Jasco system (Japan) consisting of a PU-1580 pump and a MD-1510 UV detector; a Spectra System auto-sampler AS3000 was used for sample injections, experimental data were acquired and processed by Borwin PDA and Borwin Chromatograph Software. Chromatographic separations were carried out using a Intersil ACE-3 C_8_ column (50 × 4.6 mm, 3 μm, CPS Analitica) and a security guard LiChrosorb^®^ RP-8 column (LiChroCART^®^ 4-4, 5 μm). The mobile phase consisted of water (solvent A) and acetonitrile (solvent B). The starting mixture (50% A and 50% B) persisted for 5 minutes and then in 20 minutes the mobile phase composition became 0% A, 100% B; in the following 3 min the mixture composition came back to the initial eluting system. The flow rate was set at 1 mL/min.

### 3.2. General Procedure for the Synthesis of 3,3-Trisubstituted-α,β-Unsaturated Methyl Esters

Compound **5** (1.5, 3 or 4.2 equiv), (CF_3_SO_3_)_2_Sn (1.7 equiv) and *N*-ethylpiperidine (1.5 equiv) were added to a stirred solution of appropriate ketones (1 equiv) in anhydrous solvent (DCM, DCE, THF, DMF, 3 mL) under nitrogen atmosphere and then the reaction mixture was exposed to microwave irradiation, applying conditions stated in Table 1, Table 2. After cooling, the mixture was filtered on a pad of Silica and crude products were purified by flash chromatography. Products of Table 2 were characterized by ^1^H-NMR, IR, and HPLC-UV/PAD analysis (Appendix I); experimental data were in agreement with the literature ones.

*(Z)-Methyl 3-(3-(benzyloxy)phenyl)but-2-enoate* [(*Z*)-**1a**, Table 1, entry 12]: 45 mg (74% yield). Yellow oil. Rf 0.37 (*n*-hexane/ethyl acetate 95:5); IR: 1932, 1717, 1678, 1275, 712 cm^-1^; ^1^H-NMR (CDCl_3_) δ (ppm): 2.20 (d, 3H, *J* = 1.3 Hz), 3.60 (s, 3H), 5.09 (s, 2H), 5.95 (q, 1H, *J* = 1.3 Hz), 6.85 (d, 1H, *J* = 7.6 Hz), 6.88 (t, 1H, *J* = 2.2 Hz), 6.97 (dd, 1H, *J* = 1.9, 7.8 Hz), 7.31 (t, 1H, *J* = 7.9 Hz), 7.35-7.52 (m, 5H); ^13^C-NMR (CDCl_3_) δ (ppm): 27.6, 51.5, 70.4 (t), 114.0, 114.4, 117.7, 120.0, 128.0, 128.4, 129.0, 129.2, 137.4 (s), 142.6 (s), 155.9 (s), 158.9 (s), 166.6 (s); UV: λmax = 223 nm; HPLC-UV/PAD analysis: RT = 5.2 min. 

*(E)-Methyl 3-(3-(benzyloxy)phenyl)but-2-enoate* [(*E*)-**1a**, Table 1, entry 12]: 10 mg (16% yield). Yellow oil. Rf 0.48 (*n*-hexane/ethyl acetate 95: 5); IR: 2946, 1712, 1574, 1289, 1204, 1159, 1024, 734, 693 cm^-1^; ^1^H-NMR (CDCl_3_) δ (ppm): 2.59 (d, 3H, *J* = 1.2 Hz), 3.78 (s, 3H), 5.11 (s, 2H), 6.16 (q, 1H, *J* = 1.2 Hz), 6.98-7.04 (m, 1H), 7.07-7.14 (m, 2H), 7.27-7.33 (m, 1H), 7.33-7.40 (m, 1H), 7.40-7.50 (m, 4H); ^13^C-NMR (CDCl_3_) δ (ppm): 18.5, 51.6, 70.5 (t), 113.7, 115.6, 117.3, 119.5, 128.0, 128.5, 129.1, 130.0, 137.2 (s), 144.1 (s), 156.1 (s), 159.3 (s), 167.6 (s); UV: λmax =217 and 267; HPLC-UV/PAD analysis: RT = 6.7 min. 

## 4. Conclusions

In this study we have developed an efficient (*Z*) selective HWE reaction of bis(2,2,2-trifluoroethyl) (methoxy-carbonylmethyl)-phosphonate with various aryl alkyl ketones combined with (CF_3_SO_3_)_2_Sn and N-ethylpiperidine using anhydrous DCE as solvent. These one-pot reactions can efficiently be performed under microwave conditions. The main favorable characteristics of our method are: (1) a short synthetic route; (2) a good selectivity for the (*Z*)- olefination of various ketones using the HWE reaction; (3) suitability for library synthesis. Further studies and applications of this new methodology will be reported in due course.

## Figures and Tables

**Figure:**
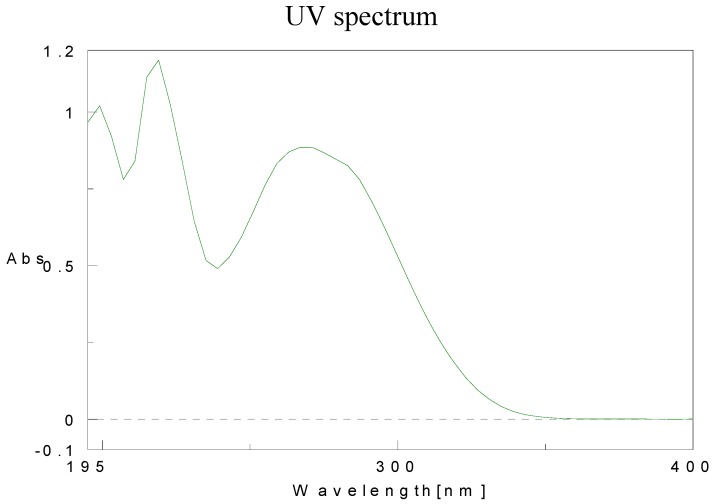
**(*Z*)-Methyl 3-(4-(benzyloxy)phenyl)but-2-enoate [(*Z*)-1b)]** Yellow oil. R*_f_* 0.18 (*n*-hexane/ethyl acetate 95:5);^ 1^H-NMR (CDCl_3_) δ (ppm): 2.20 (d, 3H, *J* = 1.4 Hz), 3.61 (s, 3H), 5.10 (s, 2H), 5.91 (q, 1H, *J* = 1.4 Hz), 6.99 (d, 2H, *J* = 8.8 Hz), 7.22 (d, 2H, *J* = 8.8 Hz), 7.33-7.39 (m, 1H), 7.39-7.50 (m, 4H); HPLC-UV/PAD analysis: RT 5.2 min.

**Figure:**
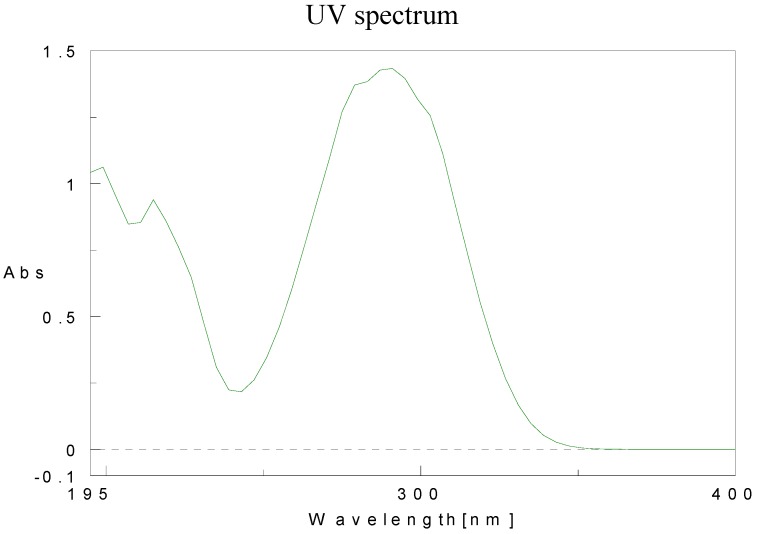
**(*E*)-Methyl 3-(4-(benzyloxy)phenyl)but-2-enoate [(*E*)-1b]** White solid (Mp = 79.4-81.0 °C). R*_f_* 0.28 (*n*-hexane/ethyl acetate 95:5); ^1^H-NMR (CDCl_3_) δ (ppm): 2.59 (d, 3H, *J* = 1.1 Hz), 3.77 (s, 3H), 5.12 (s, 2H), 6.14 (q, 1H, *J* = 1.1 Hz), 7.00 (d, 2H, *J* = 8.8 Hz), 7.34-7.39 (m, 1H), 7.40-7.50 (m, 6H); HPLC-UV/PAD analysis: RT 6.5 min.

**Figure:**
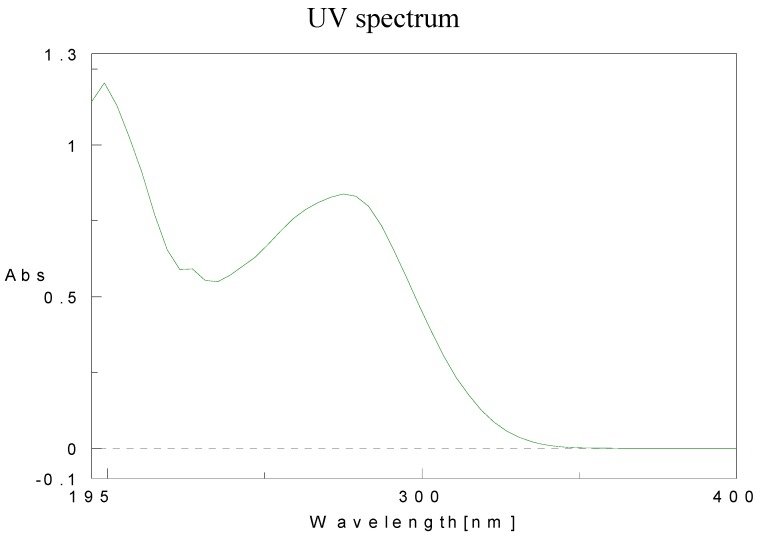
**(*Z*)-Methyl 3-([1,1'-biphenyl]-4-yl)but-2-enoate [(*Z*)-1c] **Yellow oil. R*_f_* 0.11 (*n*-hexane/ethylacetate 99:1);^ 1^H-NMR (CDCl_3_) δ (ppm): 2.25 (d, 3H, *J* = 1.4 Hz), 3.61 (s, 3H), 5.98 (q, 1H, *J* = 1.4 Hz), 7.30-7.39 (m, 3H), 7.43-7.49 (m, 2H), 7.59-7.66 (m, 4H); HPLC-UV/PAD analysis: RT 5.1 min.

**Figure:**
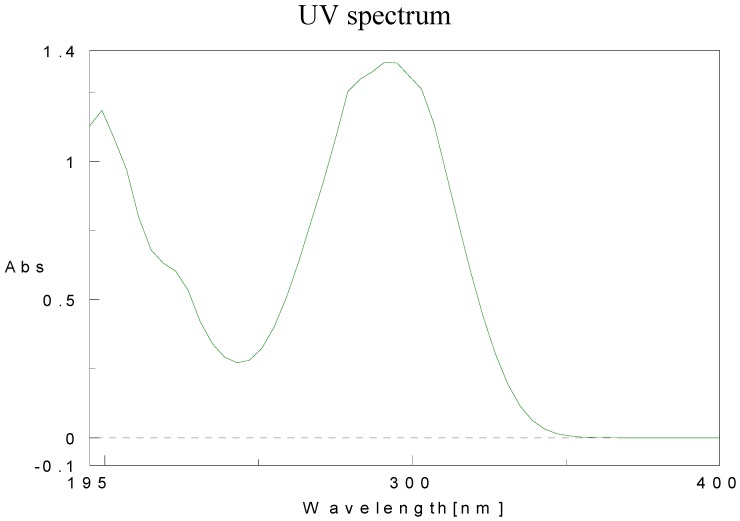
**(*E*)-Methyl 3-([1,1'-biphenyl]-4-yl)but-2-enoate [(*E*)-1c] **White solid (Mp = 135.3-137.0 °C). R*_f_* 0.19 (*n*-hexane/ethylacetate 99:1); ^1^H-NMR (CDCl_3_) δ (ppm): 2.65 (d, 3H, *J* = 1.2 Hz), 3.80 (s, 3H), 6.24 (q, 1H, *J* = 1.3 Hz), 7.37-7.42 (m, 1H), 7.45-7.51 (m, 2H), 7.57-7.67 (m, 6H); HPLC-UV/PAD analysis: RT 6.7 min.

**Figure:**
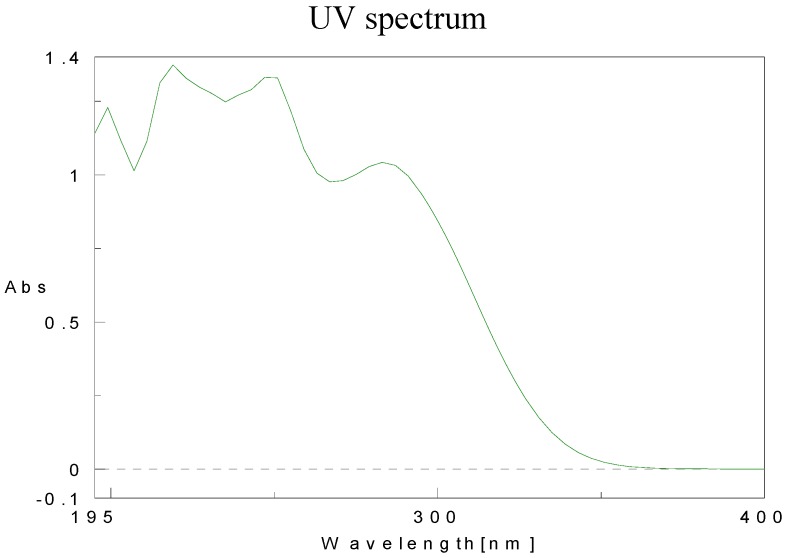
**(*Z*)-Methyl 3-(4-(phenylthio)phenyl)but-2-enoate [(*Z*)-1d]** Yellow oil. R*_f_* 0.21 (*n*-hexane/ethyl-acetate 94:6);^ 1^H-NMR (CDCl_3_) δ (ppm): 2.19 (d, 3H, *J* = 1.4 Hz), 3.60 (s, 3H), 5.94 (q, 1H, *J* = 1.4 Hz), 7.17 (d, 2H, *J* = 8.4 Hz ), 7.26-7.38 (m, 5H), 7.40-7.44 (m, 2H); HPLC-UV/PAD analysis: RT 7.0 min.

**Figure:**
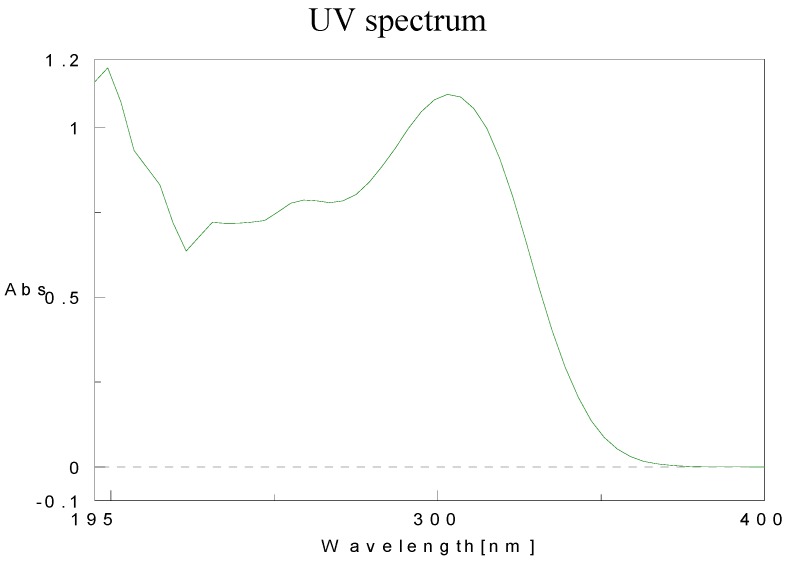
**(*E*)-Methyl 3-(4-(phenylthio)phenyl)but-2-enoate [(*E*)-1d]** Yellow oil. R*_f_* 0.28 (*n*-hexane/ethylacetate 94:6);^ 1^H-NMR (CDCl_3_) δ (ppm): 2.58 (d, 3H, *J* = 1.2 Hz), 3.77 (s, 3H), 6.15 (q, 1H, J = 1.2 Hz), 7.25-7.46 (m, 9H); HPLC-UV/PAD analysis: RT 9.2 min.

**Figure:**
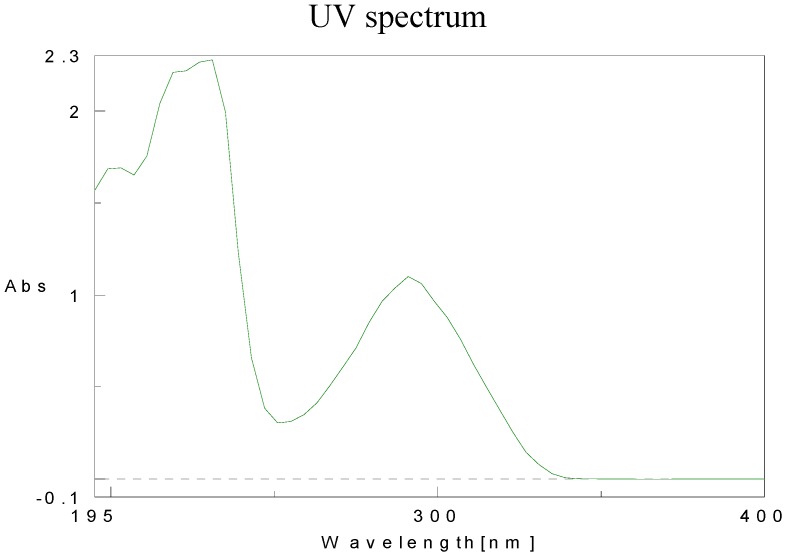
**(*Z*)-Methyl 3-(naphthalen-1-yl)but-2-enoate [(*Z*)-1e]** Yellow oil. R*_f_* 0.10 (*n*-hexane/ethylacetate 98:2);^ 1^H-NMR (CDCl_3_) δ (ppm): 2.31 (d, 3H, *J* = 1.2 Hz), 3.43 (s, 3H), 6.24 (q, 1H, *J* = 1.4 Hz), 7.21 (d, 1H, *J* =7.0Hz ), 7.60-7.67 (m, 1H), 7.72-7.83 (m, 3H), 7.95-8.05 (m, 2H); HPLC-UV/PAD analysis: RT 3.6 min.

**Figure:**
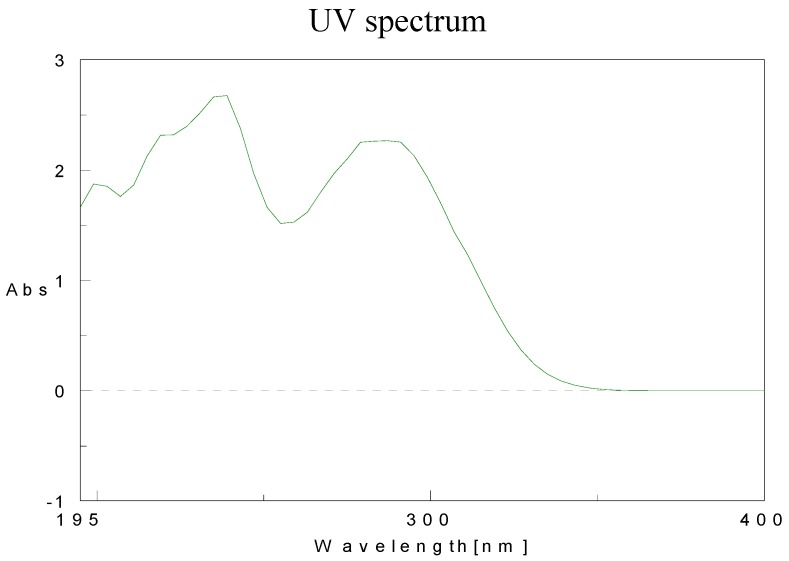
**(*E*)-Methyl 3-(naphthalen-1-yl)but-2-enoate [(*E*)-1e]** Yellow oil. R*_f_* 0.16 (*n*-hexane/ethylacetate 98:2);^ 1^H-NMR (CDCl_3_) δ (ppm): 2.66 (d, 3H, *J* = 1.1 Hz), 3.82 (s, 3H), 6.02 (q, 1H, *J* = 1.1 Hz), 7.31 (dd, 1H, *J* =7.0, 1.1 Hz ), 7.45-7.50 (m, 1H), 7.50-7.55 (m, 2H), 7.84 (d, 1H, *J* = 8.3 Hz ), 7.87-7.94 (m, 2H); HPLC-UV/PAD analysis: RT 4.7 min.

**Figure:**
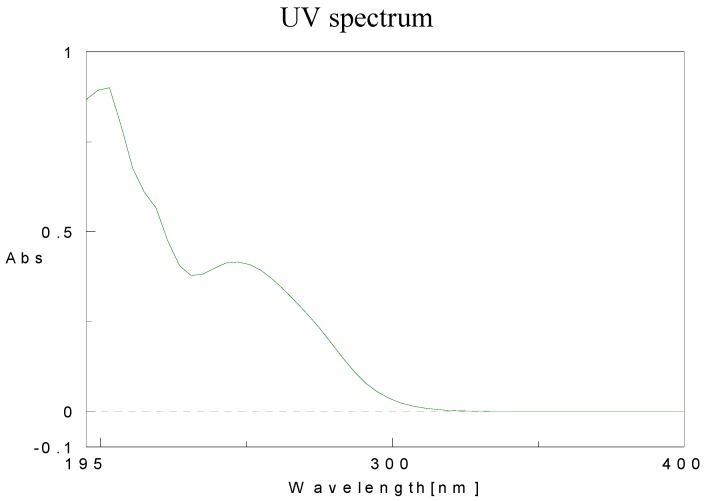
**(*Z*)-Methyl 3-phenylbut-2-enoate [(*Z*)-1f]** Yellow oil. R*_f_* 0.14 (*n*-hexane/ethylacetate 98:2);^ 1^H-NMR (CDCl_3_) δ (ppm): 2.21 (d, 3H, *J* = 1.4 Hz), 3.58 (s, 3H), 5.96 (q, 1H, *J* = 1.4 Hz), 7.21-7.26 (m, 2H), 7.31-7.42 (m, 3H); HPLC-UV/PAD analysis: RT 2.1 min.

**Figure:**
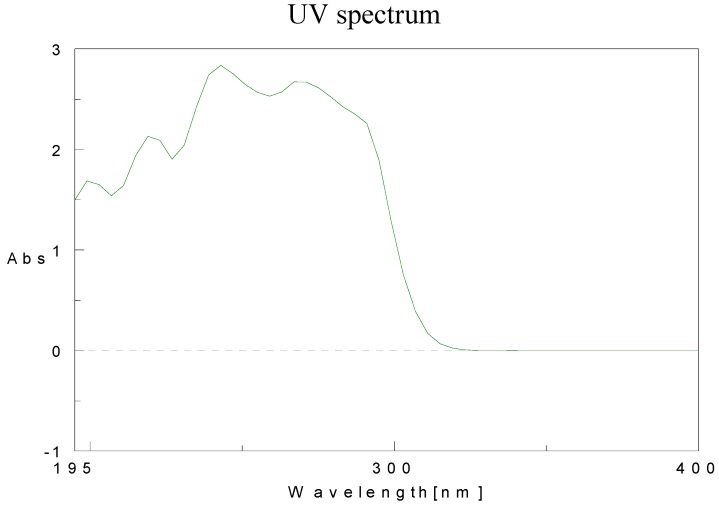
**(*E*)-Methyl 3-phenylbut-2-enoate [(*E*)-1f]** Yellow oil. R*_f_* 0.24 (*n*-hexane/ethylacetate 98:2);^ 1^H-NMR (CDCl_3_) δ (ppm): 2.61 (d, 3H, *J* = 1.3 Hz), 3.78 (s, 3H), 6.17 (q, 1H, *J* = 1.3 Hz), 7.36-7.44 (m, 3H), 7.46-7.54 (m, 2H); HPLC-UV/PAD analysis: RT 2.7 min.

**Figure:**
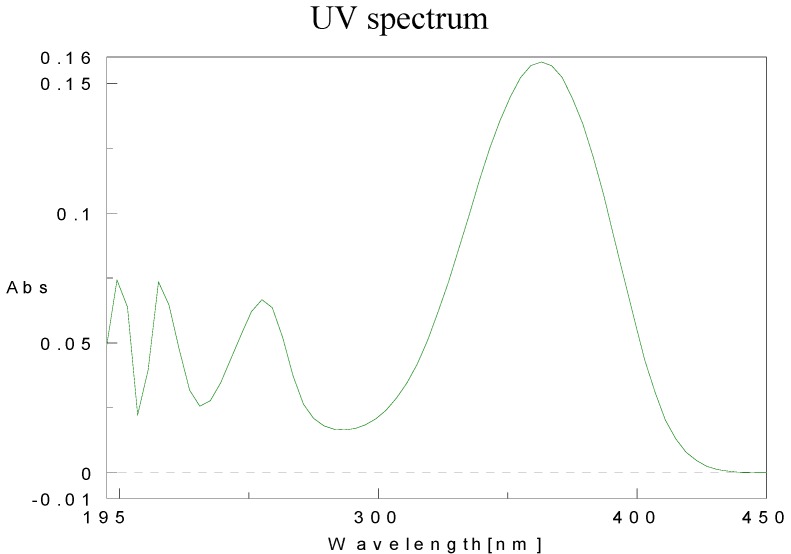
**(*Z*)-Methyl 3-(thiophen-2-yl)but-2-enoate (*Z*-1g)** Yellow solid (Mp = 88.7-90.0 °C). R*_f_* 0.21 (*n*-hexane/ethylacetate 97:3); ^1^H-NMR (CDCl_3_) δ (ppm): 2.32 (d, 3H, J =1.2 Hz), 3.71 (s, 3H), 5.88 (q, 1H, J =1.2), 7.40-7.43 (m, 2H), 7.49-7.52 (m, 1H); HPLC-UV/PAD analysis: RT 1.9 min.

**Figure:**
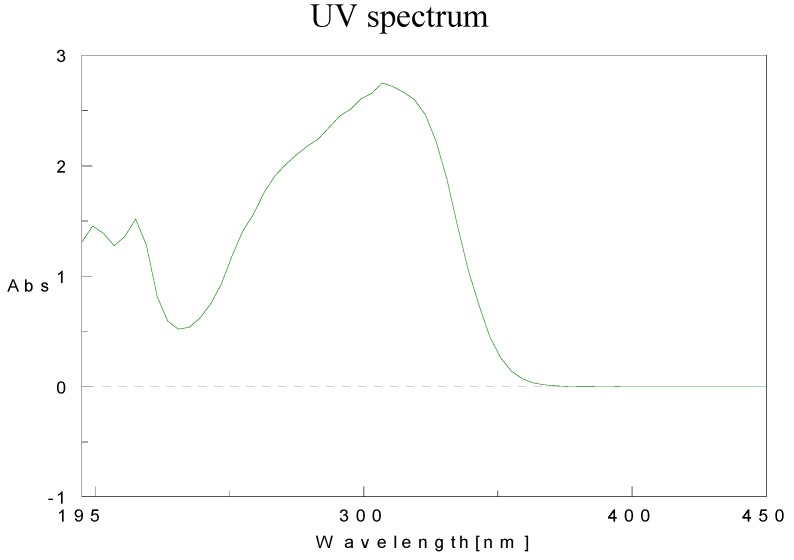
**(*E*)-Methyl 3-(thiophen-2-yl)but-2-enoate [(*E*)-1g]** Yellow oil R*_f_* 0.26 (*n*-hexane/ethylacetate 97:3); ^1^H-NMR (CDCl_3_) δ (ppm): 2.64 (d, 3H, J =1.2 Hz), 3.77 (s, 3H), 6.29 (q, 1H, J =1.2), 7.06-7.09 (m, 1H), 7.33-7.37 (m, 2H); HPLC-UV/PAD analysis: RT 2.4 min.

**Figure:**
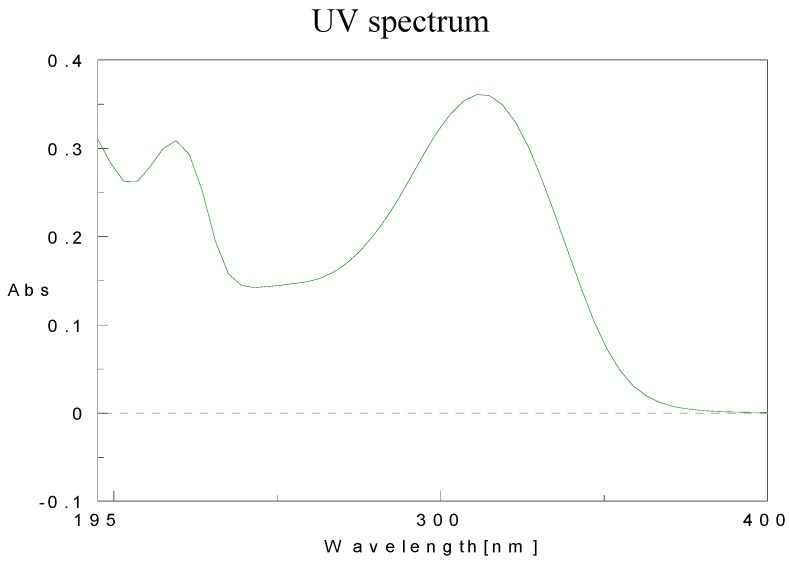
**(*Z*)-Methyl 3-(5-bromothiophen-2-yl)but-2-enoate [(*Z*)-1h]** Yellow oil R*_f_* 0.32 (*n*-hexane/ethylacetate 95:5);^ 1^H-NMR (CDCl_3_) δ (ppm): 2.30 (q, 3H, *J* = 1.2 Hz), 3.74 (s, 3H); 5.86 (d, 1H, J = 1.1 Hz), 7.09 (d, 1H, *J* =4.0), 7.26 (d, 1H, *J* =4.0); HPLC-UV/PAD analysis: RT 3.4 min.

**Figure:**
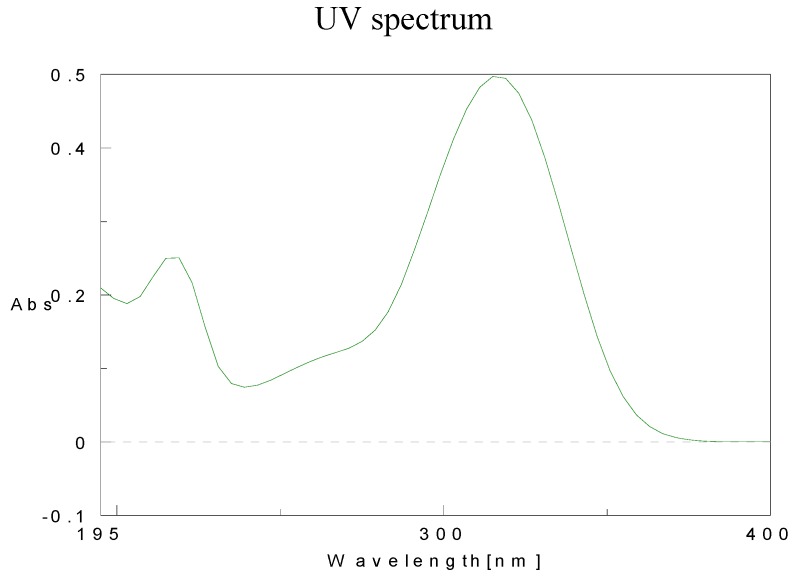
**(*E*)-Methyl 3-(5-bromothiophen-2-yl)but-2-enoate [(*E*)-1h]** Yellow oil R*_f_* 0.32 (*n*-hexane/ethylacetate 95:5);^ 1^H-NMR (CDCl_3_) δ (ppm): 2.57 (d, 3H, *J* = 1.1 Hz), 3.76 (s, 3H); 6.15 (q, 1H, J = 1.0 Hz), 7.00-7.05 (m, 2H); HPLC-UV/PAD analysis: RT 4.1 min.
